# "pp65 antigenemia and real time polymerase chain reaction (PCR) based-study to determine the prevalence of human cytomegalovirus (HCMV) in kidney donors and recipients with follow-up studies."

**DOI:** 10.1186/1743-422X-7-322

**Published:** 2010-11-16

**Authors:** Hajib N Madhavan, Moses Y Samson, Murali Ishwarya, Ramanathan Vijayakumar, Malathi Jambulingam

**Affiliations:** 1 L & T Micobiology Research Center, Sankara Nethralaya, 18, College Road, Chennai - 600 006. India; 2 Kaliappa Renal Centre, Billroth Hospitals, Chennai, India

## Abstract

**Background:**

The present study was undertaken to determine the rate of occurrence of Human cytomegalovirus (HCMV) among kidney transplant recipients and donors by application of direct detection methods and to understand HCMV infection/disease development among transplanted patients as a prospective study.

**Results:**

Peripheral blood samples collected from 76 kidney donors and 76 recipients from September 2007 to August 2009 were subjected to pp65 antigenemia and Quantitative real-time PCR (qRT-PCR) assays. Data were analyzed under Group A, B and C. Group A was further divided into sub-groups I, II, III, IV, and V for better understanding. Three, one and two donors in sub-group I, III, IV of Group A tested positive for real time PCR respectively. One recipient from group III tested positive for HCMV by qRT- PCR prior transplantation and remained positive one month post-transplantation. Three other recipients, tested negative prior to transplantation became positive a month after transplantation. Group B consisted of 18 donor-recipient pairs and one of the donor tested positive for HCMV by qRT-PCR. Eight recipients tested positive for HCMV one month after transplantation. The pp65 positivity and HCMV DNA load was high among group C recipients who mostly had symptoms of active disease. Significantly high values of pp65 antigenemia were observed among recipients of sub-group II (non-parametric chi-square test p = 0.007). Positive correlation between pp65 antigenemia and qRT-PCR value was observed. Thirty three of the recipients with disease treated with Valgancyclovir showed improved clinical outcome.

**Conclusion:**

Our study showed that a significant proportion of kidney recipients develop HCMV infection following renal transplantation in spite of the absence of HCMV among donors. pp65 antigenemia assay and qRT- PCR methods can be applied to detect HCMV among kidney donors and recipients to monitor development of disease and these assays were predicative of HCMV infection among them. Clinical resistant to valganciclovir was not observed.

## Background

Human Cytomegalovirus causes significant morbidity and mortality in immunocompromised patients, who have undergone solid organ or bone marrow transplantation [1,2]. Due to a depressed immune system, CMV-related disease may be much more aggressive in kidney transplanted patients. HCMV is one of the causes for the failure of the graft [3]. The presence of latent HCMV in both donors and recipients could be a source of HCMV infection among kidney transplant recipients [4]. It was Hughes in 2008, who showed the impact of donor recipient sero-status on CMV antigenemia in a large cohort of renal transplant recipients [3]. As donors are potential sources of HCMV infection, a positive HCMV detection in them, should prompt close monitoring of the recipients for the development of this viral disease by sensitive methods. In recent years, owing to an increase in the antiviral prophylaxis, there has been a decline in reduction in morbidity and mortality caused by this virus [5]. Since unwanted exposure to gancyclovir results in development of gancyclovir resistance, the preemptive strategy is widely followed. Though the occurrence of HCMV disease is reduced markedly among transplant recipients during the therapeutic period, the infection/disease has been reported on withdrawal of the drugs. Sensitive and specific diagnostic methods are needed not only for the early detection of HCMV infection in transplanted patient to initiate the therapy, but also to continuously monitor them if needed thereafter. Because of the latent life long HCMV infection many transplant patients secrete HCMV without any clinical disease and therefore the mere detection of HCMV does not always indicate the need for treatment. Quantitation of systemic HCMV load may provide a highly sensitive and specific method to have insight to predict which of these patients may develop HCMV disease. The present study was undertaken to determine the rate of detection of HCMV among kidney donors and recipients by application of pp65 antigenemia and quantitative real - time PCR. Investigations are still going on to determine the exact relationship between HCMV DNA and pp65 antigenemia levels. We have also studied the relative diagnostic value of real time PCR (qRT-PCR) with pp65 antigenemia. The importance of correlating the test results with clinical symptoms is given.

## Results

A total of 57 donor recipient pairs were included in the study. Based on the availability of follow-up samples, they were classified into Group A and Group B. Recipients from whom peripheral blood samples could be obtained at one month and after were classified under Group A. Mean follow up days is 92.6 days. Group A was further classified into five subgroups I-V based on the pp65 and qRT- PCR values. Recipients from whom one month post transplant sample could be obtained were classified under group B. Nineteen more unrelated donors and kidney recipients were included under group C in the study. (Table 1). In group A, sub-group I consisted of three donors positive for HCMV only by real time PCR whose copy numbers were 1, 144 and 289 copy/ml. The recipients were tested negative for HCMV before transplantation and by 30 days post transplantation they turned positive with, 2265 copies/ml, 119 copies/ml and, 25 copies/ml by real time PCR. None were positive for pp65 antigenemia.

**Table 1:** Analysis of results of tests performed for detection of HCMV with the donor-renal recipient patients

Groups	Donor/Recipient	No of pairs positive by real time PCR	No of pairs positive for pp65 antigenemia
Group A	*D+/PRR-/PSR+	3/3	0/3
			
Sub-Group I			

Sub-Group II	D-/PRR-/PSR+	26/26	15^$^/26

Sub-Group III	*D+/PRR+/PSR+	1/1	0/1

Sub-Group IV	*D+/PRR-/PSR-	2/2	0/2

Sub-Group V	D-/PRR-/PSR-	0/7	0/7

Group B	D-/PRR-/PSR+	6^#^/18	2/18
	
	D+/PPR-/PSR+	1/18	0/18

Group C	PSR+	19^^^/19	19/19
	
	D+	4/19	0/19

**In Group A**,

*- Donor (D), pre transplantation renal recipient (PRR) and post-transplant recipient (PSR) samples.

- Post transplant recipients were tested for HCMV infections by pp65 antigenemia and viraemia by real-time PCR.

- All the donors were pp65 antigenemia negative indicating that they were not infective.

- * Six donors belonging to sub-groups I, III and IV were positive for HCMV by real time - PCR with copy numbers ranging from 1 to 537 copies/ml were all negative for HCMV DNA.

**Group B **consisted of donors from whom sampling was not possible after one month

of renal transplantation.

**Group C **consisted of unrelated donors and recipients.

- # Two among the recipients were positive for pp65 antigenemia also.

^- ^ ^All 19 positive for HCMV DNA were also positive for pp65 antigenemia also.

^- $ ^All 15 patients samples positive for pp65 antigenemia were also positive for real time

PCR also.

- Eleven more were positive only by real time PCR assay.

Twenty six recipients (69.2%) whose pre-transplant samples along with the corresponding donor's blood, tested negative by both the methods used were classified under sub-Group II. But they became positive for HCMV one month after transplantation. Among the 26, 15 patients were tested positive for both pp65 antigenemia and real - time PCR. The highest copy number detected by real - time PCR was 29, 768 copies/ml and lowest was 4 copies/ml. The highest positive pp65 cell count recorded among them was 78 cells and the lowest was 3 cells. In eleven others, the blood sample was positive only by real - time PCR. The highest copy number recorded in among seven of them was 3079 copies/ml and lowest was 4 copies/ml. Mean pp65 antigenemia values/2 × 10^5 ^PBLs for this group was 13.8.

Sub-group III consisted of a single patient whose pre-transplantation sample had a real time count of 8 copies/ml which reduced to 7 copies/ml after a month of transplantation. The corresponding donor was also found positive for HCMV viremia with 11 copies/ml.

Sub-group IV consisted of two patient whose donors were tested positive for HCMV. Two donors were positive for real time PCR with 86 copies/ml and 537 copies/ml respectively. The corresponding recipient was negative for HCMV before and one month after transplantation.

Sub-Group V consisted of seven recipients (15.4%) whose donors, their pre-transplant specimens and three-month post-transplant specimens were negative for both pp65 antigenemia and real time PCR assay.

**Figure 1:**
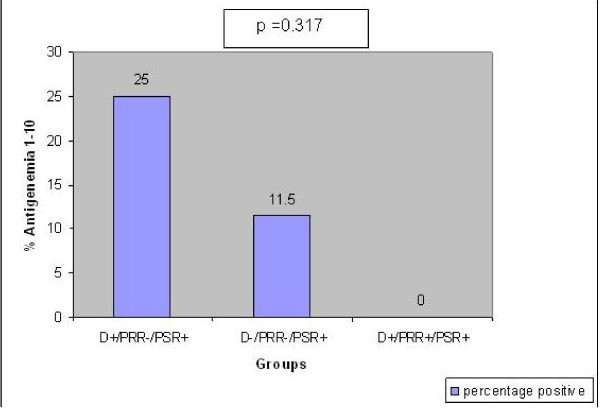
**Bar graph comparing the pp65 antigenemia values of the 3 sub-groups belonging to group 'A'**. D- Donors. PRR- Pre- Transplant Recipient specimens. PSR- Post - Transplant recipient specimens.

**Figure 2:**
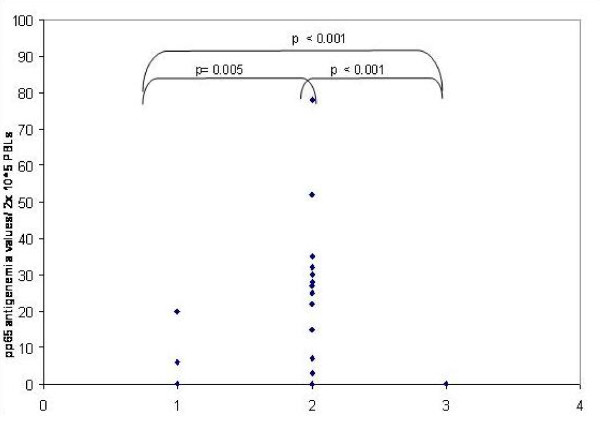
**Scatter plot comparing all 3 sub-groups of group 'A' based on pp65 antigenemia values**. 1: D+/PRR-/PSR+. 2: D-/PRR-/PSR+. 3: D+/PRR+/PSR+.

**Figure 3:**
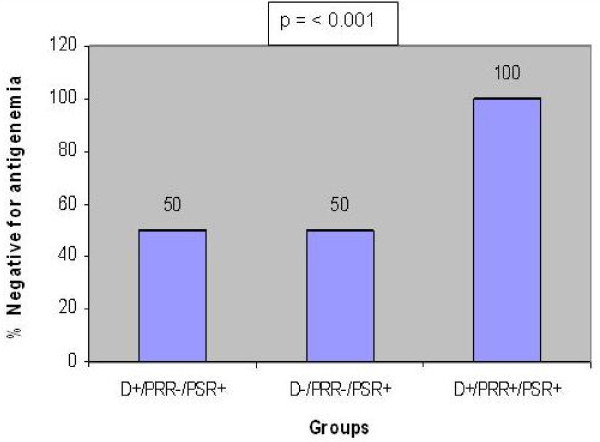
**Bar graph showing % negativity in pp65 antigenemia values among renal transplant recipients**. D- Donors. PRR- Pre- Transplant Récipient specimens. PSR- Post - Transplant récipient spécimens.

**Figure 4:**
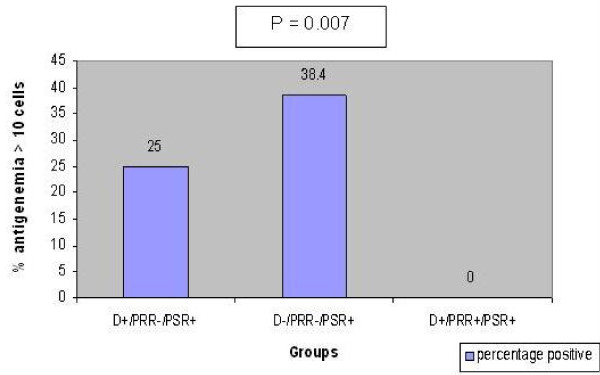
**Bar graph showing significantly high values of pp65 antigenemia among the recipients belonging to sub-group II of group 'A'**. D- Donors. PRR- Pre- Transplant Récipient specimens. PSR- Post - Transplant récipient spécimens.

There was statistical difference in the pp65 antigenemia values among the three sub-groups (non-parametric chi-square test p = 0.007, Figure 1,2,3). Significantly a high value of pp65 antigenemia was observed among the recipients belonging to sub-group II (Figure 4).

Of the (table 2) 18 donor-recipient pairs under Group B, six recipients were tested positive for HCMV DNA one month after transplantation by real-time PCR of which two were positive by pp65 antigenemia assay and 12 were negative by both real-time PCR and pp65 antigenemia assay. One donor was positive only by real-time PCR. Seventeen others were tested negative for both pp65 antigen and HCMV DNA. Of the 19 unrelated (table 2) donor-recipient pairs, all recipients were positive for the presence of both pp65 antigen and HCMV DNA the results of which were ranging from 5 - 230 cells/2 × 10^5 ^leucocytes and copy numbers ranging from 46 - 84,62,847 copies/ml respectively. Four of the donors were positive for the presence of HCMV DNA with copy numbers ranging from 14 - 458 copies/ml but none were detected by pp65 antigenemia assay.

**Table 2:** Results of HCMV performed on the 33 patients treated for the disease

Patient no	No of pp65 positive cells/2 × 10^5 ^leucocytes	HCMV DNA detected copy/ml
1	8	692

2.	20	775

3	86	8462847

4	78	3817

5	13	15386

6	25	20201

7	28	1851

8	35	647

9	25	724

10	14	1673

11	32	29768

12	38	75479

13	35	500

14	48	460

15	78	271

16	20	775

17	86	8462847

18	78	3817

19	230	30902

20	8	692

21	49	674

22	35	4849

23	7	47

24	27	337

25	3	334

26	22	7456

27	15	1411

28	40	24104

29	52	6756

30	20	2265

31	7	4937

32	0	3079

33.	32	29768

pp65 antigenemia assay and HCMV 2 × 10^5 ^leucocytes of peripheral blood and human cytomegalovirus DNA copy numbers detected/ml in the 33 renal transplant recipients who underwent valganciclovir treatment for symptomatic disease showed presence of antigenemia in 32/33 patients.

### Results on the follow up samples

In sub-group I, all three recipients were followed up to two months and became negative for HCMV viremia. Fourteen among twenty seven patients in group II were followed up. Six of them became negative. Three others remained positive with an increasing real time PCR count. Five others had varied HCMV copy numbers in the samples. One patient belonging to group III was followed up to second month and the pp65 count was raised to 37 cells and real time value was 106457 copies/ml. One among the two in-group IV, patient was followed up to three months and remained negative. Yet another patient turned positive with 9092 copies/ml during the second month and could not be followed up further. Among the seven patients in group IV, one of them was turned positive with 246 copies of HCMV/ml.

Overall a significant number of (66.6%) recipients whose donors were negative developed a post transplant HCMV infection (chi-square test p = 0.037%).

### Correlation between pp65 and real time PCR results

Among the 76 kidney donors 58 (73.68%) were positive for real time PCR and of the 56, 39 (51.0%) were positive for pp65 antigenemia with a range of 3-86 cells/2 × 10^5 ^PBML. None of them were positive for pp65 antigenemia alone.

The real time copy numbers were higher among individuals having more than 10 cells/2 × 10^5 ^PBML. The median HCMV copy number among them was 751.5 copy/ml and the mean was 276698.6 copy/ml. Among patients with equal or less than 10 cells/2 × 10^5 ^PBML, the mean copy number/ml was 2546 and the median was 119 copies/ml. A significant positive correlation was observed between the values of pp65 antigenemia and qRT-PCR (paired t test = 0.003052).

### Valgancyclovir treatment

Among the seventy-six renal transplant recipients fifty-seven were positive for HCMV after transplantation. Of the fifty-seven HCMV positives, thirty-three of them presented with symptoms of HCMV disease and were treated with Valgancyclovir (Valgan/Valcept-450 mg/tablet) and twenty-four were not treated with Valgancyclovir. **"**The exact number of pp65 positive cells/2 × 10^5 ^leucocytes PBMC and HCMV DNA copy numbers estimated/ml is presented in table 2."

## Discussion

In our study among the seventy six donor recipient pairs a significant number of the transplant recipients (66%) developed HCMV infection post transplant. Fifty seven (75%) of them were tested positive for HCMV post transplant by real time PCR while thirty seven (49%) of them were positive for HCMV by pp65 antigenemia assay. Of the seventy six donors, eleven (14.47%) of them were positive for HCMV by real time PCR while one (1.3%) of them was positive for the virus by pp65 antigenemia assay. The exact source of HCMV detected in the three recipients of sub-group I could assumed to be the donor as they were negative prior transplantation. The HCMV infection observed among twenty six recipients of sub-group II is probably due to the reactivation of the latent virus present in them or other invasive procedures that lead to transmission of HCMV. In the sub-group III of Group A since the donor's and the recipient's pre-transplantation sample were tested positive for HCMV viremia the assumption that post-transplant the recipient would develop a aggressive disease course did not occur. Results analyzed from the various groups, substantiate the fact that the HCMV DNA was present among kidney donors and the rate of detection in our study was 11.8%.

It is recommended that donors be screened for HCMV serostatus to prevent adverse outcome of HCMV disease in renal transplant patients. But the lower survival of donor positive recipient positive (D+R+) transplants rather than donor positive, recipient negative (D+R-) and the absence of relationship between CMV infection and acute cellular rejection have been reported [6-8]. In our study we observed the detection of HCMV DNA in large number of patients who along with their donor did not have HCMV DNA prior transplantation. HCMV detection in donors and recipients will be a better method to assess the status as DNA appears even before sero-conversation takes place.

Among the fifty seven HCMV positive recipients, only thirty-three (58%) of them were treated with valgancyclovir since they had symptoms of active HCMV disease, while twenty four of the recipients (42%) were not treated though they had HCMV DNA. Twenty two (39%) of the transplant recipients with HCMV infection did not develop symptomatic disease any time in the study period. Of the thirty nine recipients who were followed up, only fourteen recipients with disease were put on treatment (36%) while twenty five (64%) of the recipients were not. As a result of the increased use of Gancyclovir prophylaxis, the incidence and severity of CMV disease is significantly reduced. However, there is an increasing incidence of Gancyclovir resistant CMV infection. Therefore treatment may be restricted to symptomatic patients alone as the presence of HCMV DNA and low count of pp65 antigenemia does not always lead to development of disease process which was made evident from the study. And also, the importance of giving pre-emptive therapy to donors and transplant recipients before transplantation may not be needed.

As reported earlier, patients belonging to sub-group II of Group A had significantly more pp65 antigenemia positive count when compared to patients belonging to other groups [9]. Bossart *et al *has shown an 84% agreement between pp65 antigenemia and quantitative assays and pointed out an episode of active infection missed out by pp65 and other instance where the patient showed significant DNA-emia with very low pp65 antigenemia positivity [10]. The advantages and disadvantages of pp65 and real time PCR in diagnosing HCMV infection and disease were well described by Carini et al 2007 [11]. A median of 11 vs 30 cells pp65 positive cells were reported among asymptomatic and symptomatic patients respectively [11]. The utility of the real time PCR technique for HCMV infection and usefulness of both the technique in detection of symptomatic patients were highlighted. The cut-off of 13 cells/2 × 10^5 ^PBML cells was considered a predictor of symptomatic infection [11]. The significant positive pp65 antigenemia value quoted varies from equal or more than 5 to 30 cells. In our study predominantly a pp65 positive count of equivalent and above 10 cells/2 × 10^5 ^and also accompanied positive high DNA copy number correlated well with the disease symptoms as observed among the 33 symptomatic patients who were treated with valganciclovir. Carni et al have shown that 29.1% of symptomatic kidney transplanted patients had pp65 positivity as against 11.8% asymptomatic patients [11]. 'Minz et have reported that 14% patients with symptomatic HCMV disease tested positive by pp55 antigenemia [12]. There were 5 (14%) low-positive and 30 (86%) high-positive patients and all high-positive patients were presented with HCMV disease. According to Kim and Kwan all patients who had an HCMV antigenemia titer of higher than 50 per 400,000 leukocytes developed HCMV-related symptoms and signs during the follow-up period [13]. However, in our study, six symptomatic patients had less than 10 positive cells per 2 × 10^5^ leucocytes/PBML.

In our study, a total of 32 among 33 patients, put on treatment, tested positive for pp65 antigenemia assay. Among the 32, six of them had a pp65 count of less than 10 positive cells per 2 × 10^5 ^leucocytes. In three patients, the count ranged between 10 and 20 positive cells per 2 × 10^5 ^leucocytes. In others, the count was more than 20 with the highest being 86 positive cells per 2 × 10^5 ^leucocytes.

The prevalence of HCMV among renal transplant patients was higher (20-60%) compared to western literature [14]. A study carried out in southern part of India among renal transplant patients by application of the quantitative PCR revealed asymptomatic viremia in 60-70% of patients at each sampling point [15]. However seroprevalence data showed that 95% of healthy blood donors tested positive for anti-HCMV IgG antibodies in India [16].

In conclusion, the study observed the presence of HCMV DNA among kidney donors. The recipients develop HCMV infection even if the donors are negative for HCMV. pp65 antigenemia and real time PCR, can be applied as a qualitative measure to assess the HCMV infection among renal transplant patient and to monitor the prognosis. Treatment should be restricted to those with HCMV disease.

## Materials and methods

### Study Design

Clinical specimens were investigated at L & T Microbiology Research Centre, Vision Research Foundation, in Sankara Nethralaya, Chennai, India during September 2007 to August 2009. A total of two hundred and thirty peripheral blood samples were processed and a prospective study was carried out in three groups of donor-recipient pairs. Patients with Neutropenia, high degree of renal dysfunction, were excluded from the study.

The mean age of the recipients was 48.5 year and the female to male ratio was 2:3. The age range among recipient males was 23 to 56 and female was 23 to 59. The mean age of the donors was 36.48 and the female to male ratio was 1.3: 1. The age range among donor male was 26-60 and female was 22 to 55.

Group 'A' consisted of 39 kidney transplanted patients and the corresponding donors. One hundred and fifty six blood specimens were collected which included 117 samples from thirty-nine renal transplant recipients before and month after transplantation and their corresponding donors samples pre-transplantation. Thirty-nine more blood samples were the follow up samples collected from recipients after two months.

Group 'B' includes 18 more donors and the corresponding 18 recipients who could not be followed up one month after transplantation period. From this group thirty-six samples were collected from donors pre-transplantation and their corresponding recipients prior and one-month post transplantation.

Group 'C' included 19 donors and 19 kidney transplant recipients who were unrelated. A total of 38 blood samples were collected one-month post transplant from the recipients and pre-transplant samples were collected from the donors. Pre-transplantation samples could not be collected from the recipients.

Samples were collected in 2 ml EDTA vacutainer tubes and were transported immediately to the laboratory from Kaliappa Renal Centre, Chennai. All specimens were transported in their naïve form without any transport medium. The blood specimens were processed immediately for pp65, and real time PCR for HCMV (qRT-PCR). The study was approved by both the institute's research and ethics committee and informed consent was obtained from the donors and the renal transplant recipients who participated in the study.

### Antigenemia assay

The pp65 antigenemia assay was carried out on smears containing 2 × 10^5 ^leucocytes prepared from 5 ml of EDTA anticoagulated blood within six hours of receipt of the specimen. Smears were fixed in methanol for 10 minutes. Immunofluorescence staining was carried out on the smears using pp65 staining kit obtained from Argene SA, France. The smears were stained with mouse monoclonal antibody (Argene SA, France); 0.5% Evan's blue (Hi-media, Mumbai), was used as a counter stain. The smears were examined under fluorescent microscope (Optiphot, Nikon, Japan) with blue filter. The presence of atleast one positively stained leukocyte was defined to be positive for the assay and the result was expressed as the number of CMV pp65 positive cells per 2 × 10^5 ^leukocytes.

### DNA Extraction

Nucleic acid was extracted from 0.2 ml of EDTA - anticoagulated whole blood by using the Bioneer blood/tissue kit (Bioneer Corporation, Daejeon, Korea) according to the manufacturer's instructions. DNA was eluted from bioneer columns in a final volume of 200 μl of elution and was stored at -20°C until used. These extracted DNA samples were used for quantitative PCR assays.

### Real Time PCR Assay

Real-time PCR targeting the morphologically transforming region *mtr *II sequence was applied onto the DNA extracted from these specimens in Rotor gene Real time PCR machine (Corbett Research, Australia) using primers and thermal profile described earlier [17]. The intra-assay and inter-assay reproducibility were evaluated using triplicates of plasmid dilutions (10^1^, 10^3 ^and 10^5^) corresponding to an input of 2.5 × 10^3^, 2.5 × 10^5^, 2.5 × 10^7 ^copies/ml per reaction in the same and four independent runs respectively.

## Competing interests

The authors declare that they have no competing interests.

## Authors' contributions

VR recruited patients for the study and provided all the clinical data. IM carried out PP65 assay. YSM carried out the real time PCR assay. JM participated in the design of the study and performed the statistical analysis. HNM conceived of the study, and participated in its design and coordination. All authors read and approved the final manuscript.
